# Supplementary material for: Revisiting the vortex-core tunnelling spectroscopy in YBa2Cu3O7−δ
Source: Nat Commun. 2016 Mar 31;7:11139. doi: 10.1038/ncomms11139 (PMC4821883; doi:10.1038/ncomms11139)
Supplement: Supplementary Information — Supplementary Figures 1-2, Supplementary Note 1 [file ncomms11139-s1.pdf]

Supplementary Figures

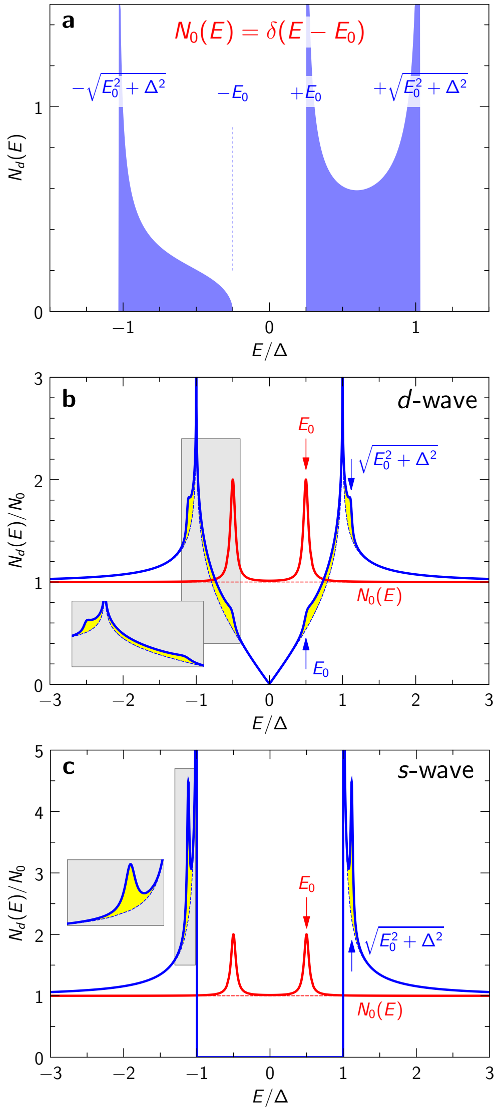

Supplementary Figure 1 | Spreading of a normal-state subgap peak by the opening of a d-wave gap. (**a**) DOS calculated with Supplementary Equation (4) for a normal-state DOS $N_0(E) = \delta(E - E_0)$ with $E_0/\Delta = 0.25$. (**b**) d-wave and (**c**) s-wave superconducting DOS (blue curves) for a normal-state DOS $N_0(E)$ given by a constant background with two symmetric peaks at $E_0 = \pm\Delta/2$ (red curves). The dashed lines correspond to a constant normal-state DOS $N_0(E) = N_0$. The yellow area highlight the spectral weight of the peaks in the superconducting DOS, and the insets show a zoom of the peak region.

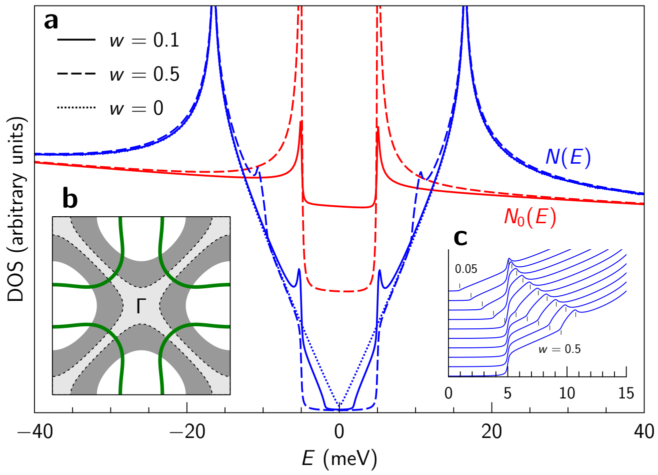

Supplementary Figure 2 | Toy model with gapped Fermi surface in the nodal regions. (**a**) Normal (red) and superconducting (blue) DOS for a model with gapped normal-state dispersion along the nodal directions. The solid lines correspond to $w = 0.1$, the dashed ones to $w = 0.5$, and the dotted line shows the usual d-wave DOS for $w = 0$. (**b**) Fermi surface (green) and gapped region around the nodal lines for $w = 0.1$ (lightgray) and $w = 0.5$ (gray). The thickness of the green line covers all momenta contributing to the DOS in **a**. (**c**) Low-energy superconducting DOS for values of w varying between 0.05 and 0.5; the curves are offset vertically. The bars indicate the energies $w\Delta$ and $[E_0^2 + (w\Delta)^2]^{1/2}$.

Supplementary Note 1

One-channel models for the spectrum of Y123 in zero field. One-channel models assume that the tunnelling conductance measured by STM on the surface of Y123 gets contributions from a single electronic band of dispersion $\xi_{\mathbf{k}} = \varepsilon_{\mathbf{k}} - \mu$, on top of a featureless background associated with transport channels of the as-grown surface and responsible for the large zero-bias conductance. In this frame of mind, the subgap peaks represent a property of the normal state which participates in pairing and is therefore gapped in the superconducting state. The dispersion $\xi_{\mathbf{k}}$ defines a normal-state density of states (DOS)

$$N_0(E) = \int \frac{d^2k}{(2\pi)^2}\delta(E - \xi_{\mathbf{k}}). \quad (1)$$

The opening of a superconducting gap changes the DOS into

$$N(E) = -\frac{1}{\pi}\int \frac{d^2k}{(2\pi)^2}\mathrm{Im}\left[\frac{E + i\Gamma + \xi_{\mathbf{k}}}{(E + i\Gamma)^2 - \xi_{\mathbf{k}}^2 - \Delta_{\mathbf{k}}^2}\right]. \quad (2)$$

The quantity in brackets is the retarded single-particle Green's function of a BCS superconductor with a gap function $\Delta_{\mathbf{k}}$, and Γ is a Dynes broadening parameter representing residual impurity scattering. We restrict ourselves to two-dimensional models.

Arbitrary isotropic normal state. We first consider a normal-state dispersion that is isotropic in the plane, $\xi_{\mathbf{k}} = \xi_{|\mathbf{k}|} \equiv \xi(k)$, but otherwise arbitrary. The Fermi surface is circular, and the details of the normal-state DOS $N_0(E)$ are controlled by the dependence of the dispersion on the modulus of $\mathbf{k}$. Any function $N_0(E)$ can in principle be parametrized by a monotonically increasing radial dispersion $\xi(k)$, by solving the implicit equation $2\pi N_0(\xi)\xi'(k) = k(\xi)$. Our goal is to express the superconducting DOS (2) in terms of the arbitrary function $N_0(E)$. For an s-wave gap, this is straightforward:

$$N_s(E) = \int_{-\infty}^{\infty} d\xi\, N_0(\xi)\,\mathrm{Im}\left[\frac{-\frac{1}{\pi}(E + i\Gamma + \xi)}{(E + i\Gamma)^2 - \xi^2 - \Delta^2}\right]. \quad (3)$$

Hence for any normal-state DOS function $N_0(E)$, the one-channel s-wave superconducting DOS for a gap magnitude Δ can be computed by performing numerically the integral in (3). If the superconducting state has d-wave symmetry, the situation is more complicated because of the angular dependence of the gap. An explicit expression can nevertheless be obtained if the gap has the functional form $\Delta\cos 2\vartheta$. Let us write $N_d(E) = -(1/\pi)\,\mathrm{Im}\,N_d(z \to E + i\Gamma)$ with

$$N_d(z) = \int \frac{d^2k}{(2\pi)^2}\frac{z + \xi(k)}{z^2 - \xi^2(k) - (\Delta\cos 2\vartheta)^2}.$$

Working in cylindrical coordinates, we note that

$$\begin{aligned}\int_0^{2\pi} d\vartheta\, &\frac{z + \xi(k)}{z^2 - \xi^2(k) - (\Delta\cos 2\vartheta)^2}\\ &= 2\pi\frac{z + \xi(k)}{\sqrt{z^2 - \xi^2(k)}\sqrt{z^2 - \xi^2(k) - \Delta^2}}\\ &= \int_0^{2\pi} d\vartheta\,\frac{z + \xi(k)}{\sqrt{z^2 - \xi^2(k)}\sqrt{z^2 - \xi^2(k) - \Delta^2}}.\end{aligned}$$

Thus $N_d(z)$ can be rewritten as a two-dimensional integral without angular momentum in the integrand, and then expressed in terms of the normal-state DOS:

$$\begin{aligned}N_d(z) &= \int \frac{d^2k}{(2\pi)^2}\frac{z + \xi(k)}{\sqrt{z^2 - \xi^2(k)}\sqrt{z^2 - \xi^2(k) - \Delta^2}}\\ &= \int_{-\infty}^{\infty} d\xi\, N_0(\xi)\frac{z + \xi}{\sqrt{z^2 - \xi^2}\sqrt{z^2 - \xi^2 - \Delta^2}}. \quad (4)\end{aligned}$$

This leads to Eq. (1) of the main text.

Supplementary Equation (4) shows that if the normal-state DOS has sharp structures, these structures are spread by the opening of the d-wave gap. To see this, take $N_0(\xi) = \delta(\xi - E_0)$ and $\Gamma = 0^+$ in (4). The resulting superconducting DOS has three square-root divergences at $E = E_0$ and $E = \pm(E_0^2 + \Delta^2)^{1/2}$ and a square-root singularity at $E = -E_0$. The spectral weight of the delta peak is spread into the intervals between these two couples of singularities [Supplementary Figure 1(a)]. The case of a normal-state DOS that is the sum of a flat background and two symmetric peaks at $E = \pm E_0$ is illustrated in Supplementary Figure 1(b) for d-wave pairing and Supplementary Figure 1(c) for s-wave pairing. While the peak structure remains visible in the s-wave case, nothing but weak features subsist in the d-wave case. In the main text it is shown that this one-channel model with an isotropic normal-state dispersion cannot explain the subgap peaks present in the STM spectra of Y123. A weak anisotropy of the dispersion will not change this conclusion qualitatively.

Strongly anisotropic normal state with nodal peaks. The failure of the one-channel isotropic model to account for the tunnelling spectrum of Y123 suggests to devise a strongly anisotropic model in which the two low-energy peaks in the normal-state DOS would be related to features localized near the nodal directions in reciprocal space. These features would not be broadened by the opening of the d-wave gap and could survive in the superconducting spectrum. Such a model is somewhat contradictory. On the one hand, we need a structure with a well-defined energy to produce prominent peaks in the normal-state. This is usually associated with a localized state, a non-dispersing band, or any phenomenon with no dispersion in momentum space. But the absence of dispersion in momentum space is the quintessence of isotropy!

We can escape this paradox if the two symmetric peaks in the normal-state DOS are due to a gap in the normal-state dispersion, which vanishes away from the nodal regions. We therefore introduce a normal-state gap $\Theta_{\mathbf{k}} = E_0\theta(w - |\cos k_x - \cos k_y|/2)$, where θ is the Heaviside function. The parameter w controls the extension of the gapped region around the nodal lines, $w = 0$ corresponding to no gap and $w = 1$ to a fully gapped Fermi surface. The modified normal-state dispersion is $\bar{\xi}_{\mathbf{k}} = \mathrm{sign}(\xi_{\mathbf{k}})(\xi_{\mathbf{k}}^2 + \Theta_{\mathbf{k}}^2)^{1/2}$. We try this model on a two-dimensional square lattice with the same dispersion as for the two-channel model presented in the Methods section, Eq. (5), but without interlayer coupling for simplicity ($t_\perp = 0$). The corresponding Fermi surface is shown in Supplementary Figure 2(b). Using the gapped dispersion $\bar{\xi}_{\mathbf{k}}$ with $E_0 = 5$ meV and a superconducting gap $\Delta_{\mathbf{k}} = \Delta(\cos k_x - \cos k_y)/2$ with $\Delta = 19$ meV, we calculate the normal and superconducting DOS using Supplementary Equations (1) and (2) and $\Gamma = 0.1$ meV. The results are displayed in Supplementary Figures 2(a) and 2(c).

The model with w = 0.1 indeed has two peaks from the normal-state DOS surviving in the superconducting DOS. A new energy scale appears below E0. This is because the superconducting gap at the edges of the gapped region is smaller than E_0. Note that our choice of the function $\Theta_{\mathbf{k}}$ with the same functional form as $\Delta_{\mathbf{k}}$ implies that the edges of the gapped region are iso-contours of $\Delta_{\mathbf{k}}$. At these edges we have simply $\Delta_{\mathbf{k}} = w\Delta = 1.9$ meV. There are no Bogoliubov quasiparticles with energy lower than $w\Delta$, such that the superconducting DOS is completely gapped below this energy. Between $w\Delta$ and E_0, the momenta outside the gapped region contribute to the DOS, which approaches the $w = 0$ curve from below, due to the missing states from the gapped regions. At E_0 we have the remnant of a normal-state square-root singularity of weight $\sim w$ due to the nearly flat dispersion of states inside the gapped region, and above E_0 the superconducting dispersion returns to conventional, and so does the DOS.

For $w = 0.5$, the DOS vanishes below E_0 because $w\Delta > E_0$ and E_0 is the smallest gap on the Fermi surface. There is no peak at E_0, the energy of the gapped states near the nodes being spread between E_0 and $[E_0^2 + (w\Delta)^2]^{1/2}$, where a weak peak remains. As seen in Supplementary Figure 2(c), the weight of the in-gap peak is independent of w, despite the fact that in the normal-state DOS this weight is proportional to w.

The model of Supplementary Figure 2 is as a proof of principle, but we consider unlikely that it explains the subgap states in Y123. The latter have a spectral weight comparable with the superconducting coherence peaks, while in Supplementary Figure 2 the weight of the in-gap peaks is tiny. More importantly, the measurements show that this weight remains the same in the superconducting and non-superconducting states, which is not the case in the model.
